# *Mycoplasma hyopneumoniae* Induces *Grap, Gadd45β*, and *secreted phosphoprotein 1* Gene Expression as Part of the Inflammatory Response in RAW264.7 Cells

**DOI:** 10.5487/TR.2009.25.3.119

**Published:** 2009-09-01

**Authors:** Mi-Hyun Hwang, Myung-Jin Choi, Seung-Chun Park

**Affiliations:** Laboratory of Veterinary Pharmacokinetics & Pharmacodynamics, College of Veterinary Medicine, Kyungpook National University, Daegu, 702-701 Korea

**Keywords:** Genefishing, *Mycoplasma hyopneumoniae*, Mycoplasmal inflammatory response, RAW264.7 cells

## Abstract

Genes related to *Mycoplasma hyopneumoniae*-induced inflammation were identified using the gene-fishing technology, an improved method for identifying differentially expressed genes (DEGs) using an annealing control primer (ACP) system in RAW264.7 cells. After treatment with *M. hyopneumo-niae*, 16 DEGs were expressed in RAW264.7 cells using a pre-screening system. Among these 16 DEGs, 11 DEGs (DEGs 1, 4, 5–10, 12-15) were selected and sequenced directly, revealing that DEG12 (Grap), DEG14 (Gadd45), and DEG15 (*secreted phosphoprotein 1*) were related to inflammatory cytokines. This is the first report that intact *M. hyopneumoniae* induces the expression of *Grap, Gadd 45β, and secreted phosphoprotein 1* in RAW264.7 cells. Subsequently, these genes may be targets for screening novel inhibitors of the mycoplasmal inflammatory response.

## NTRODUCTION

*Mycoplasma hyopneumoniae* is the etiological agent of mycoplasmal pneumonia in swine, and has high morbidity and low mortality. The bacteria colonize ciliated respiratory epithelial cells and inhibit cell function by inducing an inflammatory response. Previously, we showed that the activation of phospholipase C (PLC) causes the loss of cilia (DeBey and Ross, 1994; Park *et al.*, 2002), and our findings suggested that the receptors for pathogenic *M. hyopneumoniae* are coupled to $G_{i/o}$. Upon binding to these receptors, the G protein stimulates the PLC pathway to increase $[Ca^{2+}]_i$ through increased $Ca^{2+}$ release from the endoplasmic reticulum (ER). We also confirmed that polyclonal antibodies produced by mycoplasma vaccination do not prevent *M. hyopneumonia* - induced increases in $[Ca^{2+}]_i$ (Hwang *et al.*, 2006). In addition, pathogenic *M. hyopneumoniae* induces some pro-inflammatory cytokines, including interleukin (IL)-1β, tumor necrosis factor (TNF)-α, and IL-6 in the bronchoalveolar lavage of pigs (Asai *et al.*, 1993, 1994). Because pathogenic *M. hyopneumoniae* induces many inflammatory cytokines, leading to chronic respiratory disease with high morbidity, it is of interest to find a new screening method for the development of novel therapeutic agents *in vitro*.

Many genes must be expressed in a stage-specific manner to ensure the inflammatory response. To date, however, the pathogenic functions of only a few have been characterized. Therefore, to further understand the molecular basis of inflammation, it is necessary to identify and characterize differentially expressed genes (DEGs) in detail. To identify novel genes related to mycoplasmal inflammation, we used an annealing control primer (ACP) system to investigate mRNA expression profiles in RAW264.7 cells with or without exposure to intact *M. hyopneumoniae* (Hwang *et al.*, 2003, 2004).

## MATERIALS AND METHODS

***Mycoplasmas.*** *Pathogenic M. hyopneumoniae* was obtained from the American Type Culture Collection (ATCC 25934). The mycoplasma was cultured to the logarithmic phase in Friis medium (Friis, 1975) and harvested by centrifugation at 15,000 g for 30 min at 4°C. The mycoplasma pellets were collected and washed three times in 50 ml of cold phosphate-buffered saline

(PBS), with an additional centrifugation step at 15,000 g for 15 min. The final pellets were dispersed in PBS by passing them through a 27-gauge needle. The number of whole mycoplasma cells collected from a 200-ml culture aliquot ($3.4 \pm 1.7 \times 10^{11}$ CCU [color changing units]/ml, n = 7) was determined using serial dilutions in tubes containing Friis medium. Cell density ($1 \times 10^{11}$ CCU/ml corresponded to 0.3 mg protein/ml) was measured using the bicinchoninic acid method (Pierce, Rockford, IL).

***First strand cDNA synthesis.*** To identify first strand cDNA synthesis, total mRNA extracted from RAW264.7 cells exposed to intact *M. hyopneumoniae* (0.3 mg/ml) was used to synthesize first-strand cDNAs using reverse transcriptase. Fig. 1 shows the electrophoresis of total RNA extracted from treated and non-treated RAW264.7 cells. Reverse transcription was performed for 1.5 h at 42°C, in a final reaction volume of 20 μl containing 3 μg of purified total RNA, 4 μl of 5 × reaction buffer (Promega, Madison, WI, USA), 5 μl of dNTPs (each 2 mM), 2 μl of 10 μM dT-ACP1 (5'-CGT GAA TGC TGC GAC TAC GAT III IIT (18)-3'), 0.5 μl of RNasin® RNase Inhibitor (40 U/μl; Promega), and 1 μl of Moloney murine leukemia virus reverse transcriptase (MMLV-RT, 200 U/μl; Promega). First-strand cDNA was diluted by the addition of 80 μl of ultra-purified water for GeneFishing™ PCR, and stored at -20°C until further use.

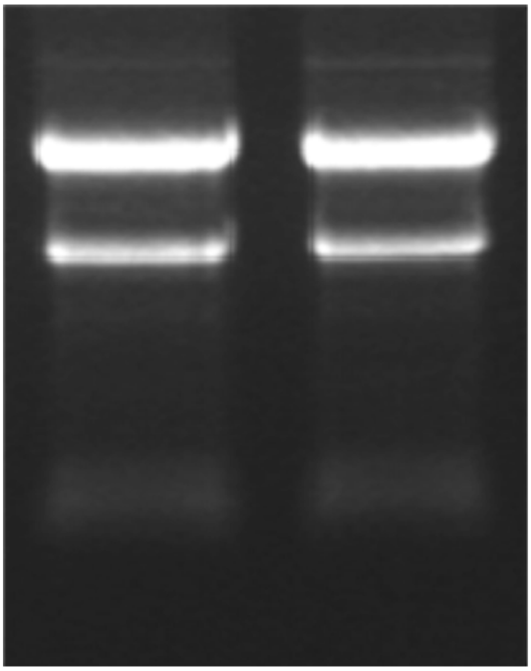

**Fig. 1.** Electrophoresis of total RNA extracted from RAW264.7 cells non-treated (A) or treated (B) with intact pathogenic *M. hyopnumoniae* (300 μg/ml) for 18 hours.

***ACP-based GeneFishing™ PCR.*** DEGs were screened using an ACP-based PCR method (Kim *et al.*, 2004) using GeneFishing™ DEG kits (Seegene, Seoul, Korea). Briefly, second-strand cDNA was synthesized at 50°C during one cycle of first-stage PCR in a final reaction volume of 20 μl containing 3~5 μl (about 50 ng) of diluted first-strand cDNA, 1 μl of dT-ACP2 (10 μM), 1 μl of 10 μM arbitrary ACP, and 10 μl of 2 × Master Mix (Seegene). The PCR protocol for second-strand synthesis consisted of 94°C for 1 min, followed by 50°C for 3 min, and 72°C for 1 min. After second-strand DNA synthesis was completed, the second-stage PCR amplification protocol consisted of 40 cycles of 94°C for 40 s, followed by 65°C for 40 s, and 72°C for 40 s, with a final 5-min extension at 72°C. The amplified PCR products were separated on 2% agarose gels stained with ethidium bromide.

***Cloning and direct sequencing.*** The differentially expressed bands were re-amplified and extracted from the gel using a GENCLEAN® II Kit (Q-Bio gene, Carlsbad, CA, USA), and sequenced (one end sequencing) directly using an ABI PRISM® 3100-Avant Genetic Analyzer (Applied Biosystems, Foster City, CA, USA) using universal primer (5'-GTC TAC CAG GCA TTC GCT TCA T-3'). The sequences were analyzed using BLASTX search program at the National Center for Biotechnology Information (NCBI) GenBank.

## RESULTS

To identify DEGs related to the inflammatory response by intact pathogenic *M. hyopneumoniae* in RAW264.7 cells, we compared mRNA expression profiles in RAW264.7 cells with or without exposure to intact *M. hyopneumoniae* for 18 h. Total RNAs were isolated from treated and non-treated cells and the RNA concentration was 0.69 and 0.72 μg/ml in the non-treated and treated RAW264.7 cells, respectively, and it was used for the first-strand cDNA synthesis (Fig. 1). Using 20 arbitrary ACP primers for the first-strand cDNA from intact form-treated cells, DEGs were screened using an ACP-based PCR method (Kim *et al.*, 2004b) using GeneFishing™ DEG kits (Seegene, Seoul, Korea).

From the differential expression of mRNA fragments observed on agarose gels, it was found 16 DEGs from the non-treated and treated RAW264.7 cells using the GeneFishing DEG pre-screening system. Among them, 11 genes (DEG 2, 3, 4, 5, 6, 8, 9, 10, 11, 12, and 16)

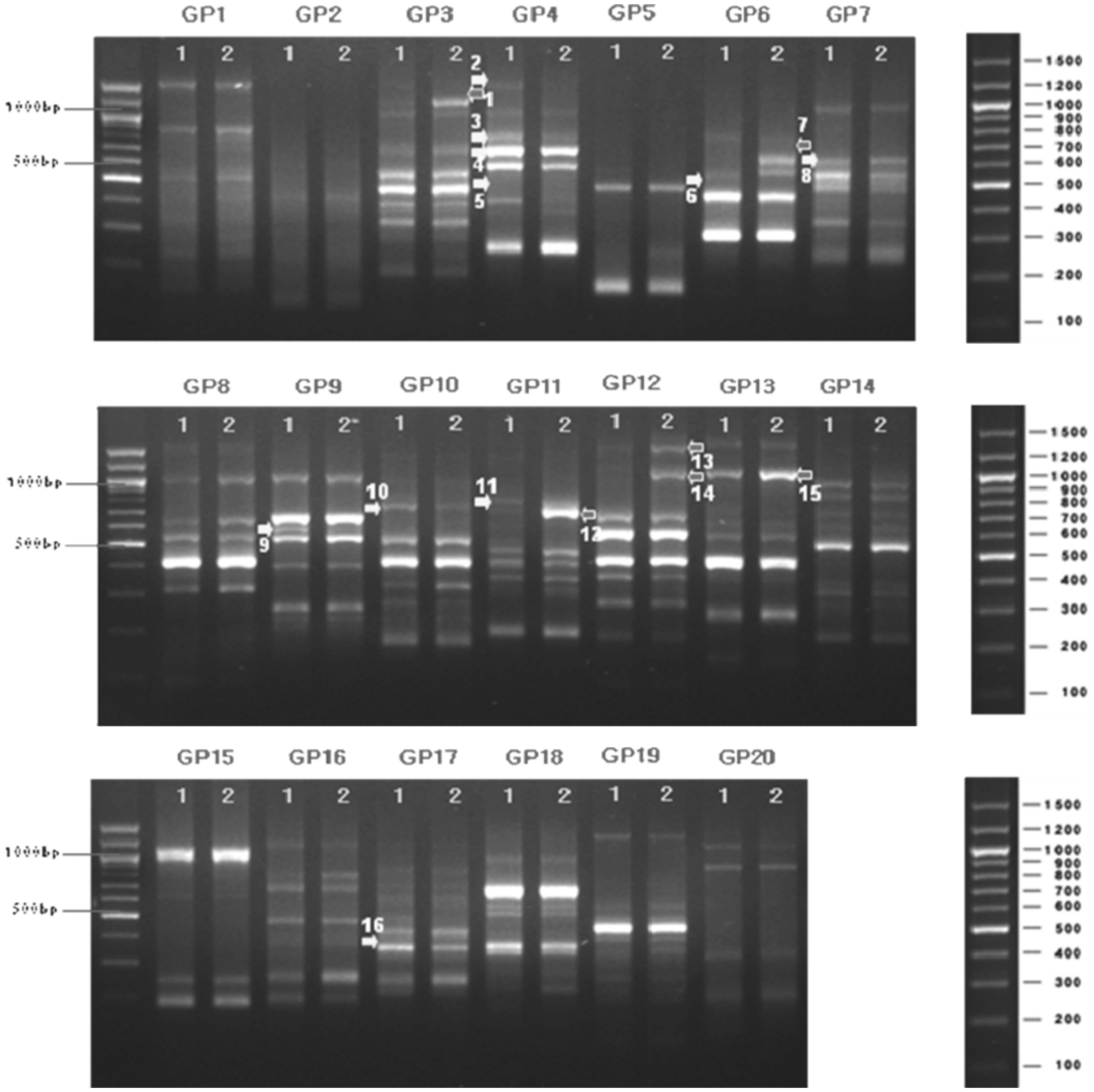

**Fig. 2.** Results of genefishing PCR for the identification of differentially expressed genes (DEGs) in RAW264.7 cells non-treated (1) or treated (2) with intact pathogenic *M. hyopnumoniae* (300 μg/ml) for 18 hours. Two-stage PCR amplification was performed using 1 of the 3 arbitrary 10-mer ACPs. Molecular weight is indicated by a 100-bp size marker generated using the Forever 100-bp Ladder Personalizer.

were down-regulated and 6 genes (DEG 1, 7, 12, 13, 14, and 15) were up-regulated in *M. hyopneumoniae*-treated RAW264.7 cells (Fig. 2).

11 obvious bands were selected to identify DEGs related to the inflammatory response in RAW264.7 cells (Fig. 3). The DEGs were re-amplified and extracted from the gel and directly sequenced by using kit described above. Sequence analysis showed that nine DEGs were known genes (Table 1). Although DEG 6 and DEG 9 had not been characterized, their full-length cDNA or expressed sequence tag (EST) sequence had been deposited in the National Center for Biotechnology Information (NCBI) database (http://www.ncbi.nlm.nih.gov/; Table 1).

## DISCUSSION

To investigate changes of gene expression associated with infection of RAW264.7 cells with *M. hyopneumoniae*, global gene expression analysis was performed

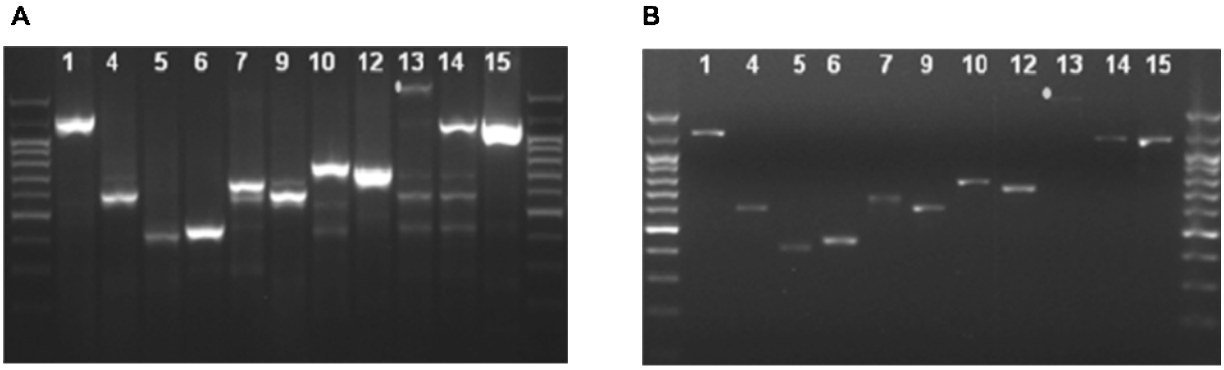

**Fig. 3.** Re-amplification (A) and gel re-extraction (B) of 11 DEG bands selected in Fig. 2. Bands from the secondary extraction were sequenced directly and the results were summarized in Table 1.

**Table 1.** Sequence alignment of differentially expressed genes (DEGs) with known full-length cDNA or expressed sequence tag (EST)

| DEG No. | GenBank accession No. | Sequence homology search |
|---|---|---|
| DEG1 | NM_133847 | *Mus musculus* transmembrane 9 superfamily protein member 4 (Tm9sf4), mRNA length = 3896 |
| DEG4 | BC042442 | *Mus musculus* SWI/SNF-related, matrix-associated actin-dependent regulator of chromatin, subfamily a, containing DEAD/H box 1-', mRNA (cDNA clone MGC:25561 IMAGE:3980726), complete cds length = 4193 |
| DEG5 | AK034007 | *Mus musculus* adult male diencephalon cDNA, RIKEN full-length enriched library, clone: 9330140A08 product: unknown EST, full insert sequence length = 2225 |
| DEG6 | BC027816 | *Mus musculus* cDNA clone IMAGE:5253491, partial cds length = 1138 |
| DEG7 | BC034513 | *Mus musculus* polo-like kinase 2 (Drosophila), mRNA (cDNA clone MGC: 7061 IMAGE: 3156743), complete cds length = 2804 |
| DEG9 | BC020382 | *Mus musculus* cDNA clone IMAGE: 3582855 length = 1654 |
| DEG10 | BC079903 | *Mus musculus* H2A histone family, member Z, mRNA (cDNA clone MGC: 96771 IMAGE: 30622912), complete cds length = 912 |
| DEG12 | NM_027817 | *Mus musculus* GRB2-related adaptor protein (*Grap*), mRNA length = 1600 |
| DEG13 | M20617 | *Mus musculus* ornithine decarboxylase mRNA, complete cds length = 1728 |
| DEG14 | AB021884 | *Mus musculus* mRNA for oncostatin M-inducible protein OIG37, complete cds length = 1007 |
| DEG15 | BC057858 | *Mus musculus secreted phosphoprotein 1*, mRNA (cDNA clone MGC: 68254 IMAGE: 5294167), complete cds length = 1403 |

using the ACP-based differential display RT-PCR (GeneFishing DEG screening technology). Olignucletide and cDNA microarray methods can be used for large scale gene expression analysis of thousands of genes at a time, however, real-time RT-PCR assay is more accurate quantitative analysis of small numbers of candidate genes. The ACP-based RT-PCR technology which has recently been developed has provided new insights into the efficacious analysis of gene expression between tissues and cell lines. This method substantially improved the specificity and sensitivity of PCR through elimination of false positives and poor reproducibility of previous DEG discovery methods such as cDNA microarray, thereby allowing real products to be amplified (Hwang *et al.*, 2003; Kim *et al.*, 2004b).

After treatment with intact *M. hyopneumoniae*, DEG1, DEG7, DEG12, DEG13, DEG14, and DEG15 were highly expressed. Of these DEGs, EST and sequence analyses suggested that DEG12, DEG14, and DEG15 are isomers of Grap, growth arrest and DNA-damage-inducible 45 gamma (Gadd45$\gamma$), and secreted phosphoprotein 1 or osteopontin, respectively. The remaining DEGs observed during the inflammatory response have not been characterized in detail.

Shiraiwa *et al.* (2004) reported that the Grap gene might have a role in the pathogenesis of Sjögren's syndrome, an autoimmune disease in which the body's immune system mistakenly attacks its own moisture-producing glands (Shiraiwa *et al.*, 2004). Shen *et al.* (2002) showed that Grap inhibited cell proliferation, IL-2 production, and c-fos induction in response to mitogenic T-cell receptor (TCR) stimulation mediated by the Ras/Erk pathway, but not by JNK/SAPK or p38 mitogen-activated protein (MAP) kinase pathways (Shen *et*

*al.*, 2002).

To our knowledge, no data are available regarding the role of Grap in bacterial pathogenesis. Fig. 1 show that Grap was strongly expressed in *M. hyopneumoniae*-treated RAW264.7 cells, suggesting that Grap expression is a good indicator for the initial screening of bioactive materials to prevent *M. hyopneumoniae* infection.

Unlike Grap, Gadd45β activates TCR signaling or inflammatory cytokines in T cells, such as IL-12 and IL-18, by activating the p38 and JNK/SAPK MAP kinase pathways (Lu *et al.*, 2001). Gadd45β-deficient mice showed an impaired T helper type 1 response to *Listeria monocytogenes* infection (Lu *et al.*, 2004a). Based on these reports, Gadd45β may also be activated in the acute inflammatory response to bacterial infection, which is consistent with our results showing that pathogenic *M. hyopneumoniae* activates the Gadd45β gene in RAW264.7 cells.

Mycoplamsa could induce the up-regulation of secreted phosphoprotein 1 (Osteopontin). Osteopontin plays an important role in lung pathologies, such as abnormal pulmonary granuloma formation (O'Regan *et al.*, 2001). In addition, Serlin *et al.* (2006) reported that IL-1β induces osteopontin expression in pulmonary fibroblasts (Serlin *et al.*, 2006). Recently, Kohan *et al.* (2007) reported that osteopontin expression in the lungs increased in a murine model of allergen-induced chronic airway remodeling, suggesting a role for this cytokine in airway remodeling in asthma (Kohan *et al.*, 2007). In a previous report (Hwang *et al.*, 2008b), it was found that intact pathogenic *M. hyopneumoniae* induced the transcription of pro-inflammatory cytokines, such as cyclooxygenase (COX)-2, TNF-α, IL-1β , IL-6, and inducible NO synthase (iNOS) in RAW264.7 cells (Hwang *et al.,* 2008b). In the same cells, mycoplasma induced osteopontin gene expression, suggesting that IL-1β induces the osteopontin gene, as do pulmonary fibroblasts. Therefore, this gene may also be a candidate for screening for inhibitors of *M. hyopneumoniae* infection.

In conclusion, we have searched for differentially expressed anti-inflammatory genes that are induced in RAW264.7 cells after exposure to intact *M. hyopneumoniae*. Our findings suggest that DEG 12 (*Grap*), DEG 14 (*Gadd45β*), and DEG 15 (*secreted phosphoprotein 1*) are attractive novel target genes for screening anti-inflammatory agents in *M. hyopneumoniae*-treated RAW264.7 cells.

## ACKNOWLEDGMENTS

This work was supported in part by the Korea Research Foundation Grant funded by the Korean Government (KRF-2008-521-E00146), and this work supported in part by Ministry of Knowledge Economy (MKE) through the Center for Traditional Microorganism Resources (TMR) at Keimyung University.
